# Integrated Filter Design for Analog Field Mill Sensor Interface

**DOI:** 10.3390/s23073688

**Published:** 2023-04-02

**Authors:** Zoi Agorastou, Anastasios Michailidis, Aikaterini Lemonou, Rafaela Themeli, Thomas Noulis, Stylianos Siskos

**Affiliations:** Electronics Laboratory, Physics Department, Aristotle University of Thessaloniki, 54124 Thessaloniki, Greece

**Keywords:** integrated bandpass filter, noise filtering, Operational Transconductance Amplifiers, passive element replacement, leapfrog, analog sensor interface, electric field mill

## Abstract

The design process of an integrated bandpass filter targeted for the noise filtering stage of the synchronous demodulation unit of an electric field mill sensor interface is presented. The purpose of this study of filter integration techniques is to avoid the challenging and, in some cases, impossible passive element integration process and to incorporate the final filter design in an entirely integrated field mill sensing system with superior performance and an optimized silicon-to-cost ratio. Four different CMOS filter implementations in the 0.18 μm process of XFAB, using OTA (Operational Transconductance Amplifier)-based configurations for passive element replacement in cascaded filter topologies and leapfrog techniques, are compared in terms of noise performance, total harmonic distortion, dynamic range, and power consumption, as well as in terms of integrability, silicon area, and performance degradation at process corners/mismatches. The optimum filter design performance-wise and process-wise is included in the final design of the integrated analog readout of a field mill sensor, and post-layout simulation results of the total circuit are presented.

## 1. Introduction

Electric field measurements are an important aspect in the assessment of the electric environment under the HVDC transmission lines [1,2] in aircraft measurements where the profiling of the fair-weather electric field provides a valuable understanding of the surrounding environment during flights [3], as well as in the study of meteorological and atmospheric phenomena and especially weather forecasting [4]. In weather forecasting, the ability to measure both fair and foul weather electric fields can be utilized to predict an imminent thunderstorm or other extreme weather phenomena [5,6,7].

Employing the principle of charge induction, the electric field mill sensor creates the effect of a pseudo-AC field by periodically exposing its sensing electrodes to the incident DC electric field. The periodical charge accumulation on the surface of the electrodes results in a current signal with an amplitude proportional to the amplitude of the electric field, thereby the processing of this signal, i.e., conversion into a voltage signal, amplification, filtering, and, finally, synchronous demodulation, can provide an accurate estimation of the external electric field [8].

While the majority of applications employing the field mill sensor utilize discrete components to compose the analog readout electronics, an ASIC (Application-Specific Integrated Circuit) sensor interface implementation offers specific benefits; besides the cost-effectiveness and the reduced size that characterizes integrated circuits, custom design feasibility of the preamplification stage allows for a combination of the typically counteractive low-noise and low-power design features, which result in an amplifier design tailored to satisfy the application’s needs. An additional advantage of integration can be more prominent in MEMS implementations of electric field sensors that also employ the charge induction principle of operation, therefore requiring a similar approach to the field mill in signal processing [9,10]. In MEMS, the proximity of the semiconductor detector to the readout electronics and, particularly, the preamplification stage minimizes the leakage currents caused by the sensing electrodes or from the stray capacitances of the necessary wire connections [5]. This leads to an improved accuracy in measurements, as well as a greater sensitivity due to the decreased induced noise. Finally, system integration allows for the simultaneous design of both analog and digital blocks for the signal processing, which is a desirable feature in this application since digital processing of the acquired information can be beneficial for error corrections and further data analysis.

A prototype IC sensor interface has already been developed in a previous work [11], where emphasis was placed on the noise optimization of the preamplification stage, which consisted of a custom designed integrated operational amplifier. The integrated preamplifier design in [11] had an input-referred spot noise of 66.7 nV/√Hz at 25.5 Hz which is comparable or, in some cases, outperforms discrete IC op-amps, while simultaneously offering the benefits of integrated design. The synchronous demodulation stage of the proposed interface is designed employing a switch-based multiplier, which allows for full design integration. An additional requirement of this analog interface is the low power consumption of its electronics, which provides the possibility to supply the system for long periods of time employing energy harvesting methods. Energy harvesting to supply the field mill operation is feasible, since the sensor is typically placed outdoors, where, during the day, solar energy is abundant.

For the realization of this interface, a bandpass filter is necessary to complement the phase sensitive detection stage in terms of noise reduction. The field mill sensor’s induced signal typically belongs to the low frequency domain (order of tens to hundreds of Hz); therefore, the filter’s passive components should have high values. For the filtering stage of the field mill interface presented in [11], a multiple feedback narrow bandpass filter was designed, which required the employment of several passive components. In ASICs, bulky passive components are challenging to integrate; thus, several design techniques are adopted to circumvent this issue. Passive element replacement by OTA-based or CCII-based simulator structures is a preferred method in filter integration [12,13], due to its non-complex design logic and its adaptability to a variety of application specifications. Filter integration is also beneficial in that it allows design optimization in terms of noise, power consumption, and area. The proposed interface implementation, which utilizes the fact that the frequency of the induced sensing signal is known to filter out unwanted noise components, has not been addressed in the field mill sensing and interfacing literature. In addition, this implementation does not require an analog-to-digital converter or a microcontroller unit for data processing since the output DC signal contains information on both the amplitude and the polarity of the field.

In this work, three different OTA-based implementations for passive element replacement in cascaded configurations of a bandpass filter, as well as an implementation based on the leapfrog technique, with an indicative passband in the low frequency domain are thoroughly simulated and compared, as to determine the optimum design for this application. Advanced simulations are conducted to the optimum filter design, which is adjusted to suit the specifications of the field mill sensor under study, to clearly demonstrate its proper operation. Simulations of the total front-end circuit, with special focus on the frequency spectrum behavior, are presented; the aim is to clearly showcase the effect of the filter and the total interface in general on the noise minimization and the signal amplitude extraction.

This paper is structured as follows: in Section 2, a description of the proposed field mill sensor interface is provided, and a brief frequency-domain analysis of each stage is included; Section 3 consists of an extensive comparative analysis of the bandpass filter designs, as well as the advanced simulations conducted on the optimum filter design; simulation results of the total analog front-end of the field mill are presented in Section 4, while in Section 5 the final conclusions of this work are discussed.

## 2. Electric Field Mill Sensor Interface and Specifications

**Figure 1:**
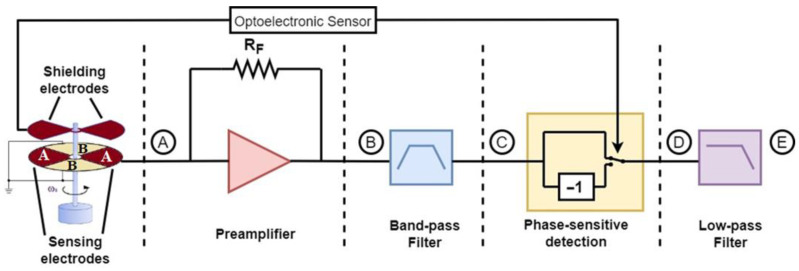
Block diagram of the field mill sensor readout system.

Figure 1 depicts the block diagram of the analog sensor interface. The readout front-end of an electric field mill sensor consists of a preamplification stage, typically a transimpedance amplifier, which converts the induced current into a voltage signal. The amplifier should introduce minimum noise for the interface to provide enhanced sensitivity.

As it was extensively described in [11], the proposed sensor interface includes a simplified realization of synchronous demodulation, which utilizes a bandpass filter to reduce noise and a modified switch-based multiplier for phase-sensitive detection, a necessary stage to extract the amplitude as well as the polarity of the field. To realize phase-sensitive detection, an optoelectronic or an inductive sensor is employed, which provides a reference signal that indicates the position of the rotating electrodes in relation to the sensing electrodes.

This implementation essentially imitates the logic adopted in the synchronous demodulation of sensors that have undergone AC excitation, and it was selected due to specific features of the field mill sensor; that is, the induced sensor signal has a known and easily regulated frequency that depends exclusively on the sensor design, i.e., the motor frequency and the number of vanes. This fact translates to a possibility of noise filtering using a narrow bandpass filter. The switch-based multiplier was preferred over an analog multiplier implementation, e.g., based on the Gilbert cell, due to its design simplicity and integrability.

In the case of the field mill sensor, a pseudo-AC electric field is created from the source DC electric field by alternatively covering and exposing the sector-shaped sensing electrodes of the field mill to the external field using a grounded rotating shutter. The shutter, which consists of sector-shaped vanes, rotates around an axis with the use of a brushless DC motor. In this manner, the alternate charge accumulation and expulsion on the surface of the sensing electrodes for an electric field *E* result in a current that is given by [8]:

(1) $$i_{A}\left(t\right)=\{\begin{array}{c} \frac{1}{2}n\varepsilon_{0}\omega_{r}\left(R^{2}-r^{2}\right)Ε\;\;\;\;\;\;\;\;\;0\leq t\leq \frac{T}{2}\;\;\;\\ -\frac{1}{2}n\varepsilon_{0}\omega_{r}\left(R^{2}-r^{2}\right)Ε\;\;\;\;\;\;\frac{T}{2}\leq t\leq T \end{array}$$

where $T=\frac{2\pi }{n\omega_{r}}$, *ω_r_* = 2*πf_r_* is the rotation speed; *n* is the number of vanes of the sensing electrodes; *R* is the external radius; and *r* is the internal radius of the sensing electrodes’ circle. Since the motor frequency is limited to a few tens of Hz due to design constraints and the number of vanes typically ranges from 2 to 8, the sensor signal ranges from a few tens to a few hundreds of Hz.

In real conditions the induced current is not a square waveform but rather a sine-like waveform [8]. Therefore, the output of the preamplifier (point B in Figure 1) can be described by:

(2) $$u_{D}\left(t\right)=A\sin n\omega_{r}t$$

where A∝E.

**Figure 2:**
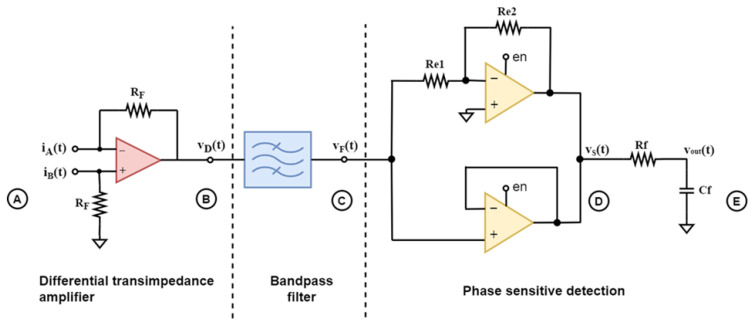
Sensor interface.

Figure 2 depicts a more descriptive schematic of the interface circuitry. At the final field mill sensor design, two sets of electrodes were employed to conduct differential measurement. This is achieved by not connecting the second set of electrodes (set B) to the ground as shown in Figure 1 but using them as sensing electrodes instead. From Figure 1, it is observed that when set A of the sensing electrodes is completely covered, set B is completely exposed and vice versa. The first stage is a differential transimpedance amplifier that converts the induced current from two sets of electrodes into a single-ended voltage signal. The two sets are alternatively covered and exposed by the shielding electrodes, resulting in two current signals, i_A_(t) and i_B_(t), with a phase difference of 180°.

Since the frequency of the derived signal is predetermined and known, a bandpass filter with a passband that contains this frequency is included to filter out the unwanted frequency components, such as slow linear trends, odd harmonics caused by the vane shape of the sensing electrodes [14], spurious signals, etc. The passband of the filter is determined by the sensor parameters and, ideally, should have a center frequency around the expected signal frequency, which is given by:

(3) $$f_{s}=nf_{r}$$

The passband of the filter should additionally have a constant gain around the expected signal frequency, due to the motor’s slightly unstable rotation. Additionally, its stopband should include most of the odd harmonics of the fundamental signal frequency to effectively reject the induced noise.

After filtering, a square-wave based synchronous demodulator is employed, which essentially implements a process that is mathematically equivalent to the multiplication of the sensor signal and the reference signal extracted from the optoelectronic sensor, which acts as a zero-crossing detector. The latter produces a square wave signal that reveals the state of exposure of the sensing electrodes surface. Instead of a switch that is controlled by the reference signal (such as in Figure 1), the synchronous demodulator employs a buffer and an inverting amplifier (gain of 1), which are enabled alternatively according to the state of the reference signal, *en* (if *en* = “*HIGH*”, the buffer is enabled, and if *en* = “*LOW*”, the inverting amplifier is enabled).

The raw sensor signal and the output signal of the optoelectronic sensor have the same frequency and, according to theory, have a phase difference of either 0° or 180° based on the polarity of the electric field. If filtered in the same manner, the initial phase difference between these signals is maintained, and polarity extraction is feasible by using the filtered optoelectronic sensor signal, *en*, as the control signal that activates/deactivates the buffer and the non-inverting amplifier.

According to Fourier analysis, the spectrum of a square wave consists of sinusoids at the odd harmonics of the square wave fundamental frequency—which is equal to the frequency of the sensor signal. Let’s assume the filtered optoelectronic sensor signal is given by:

(4) $$en=\overset{\infty }{\underset{m=1,\;\;3,5,\ldots }{\sum }}\frac{4}{m\pi }\sin\;\left(2\pi mf_{s}t\right)=\frac{4}{\pi }\sin\;\left(2\pi f_{s}t\right)+\frac{4}{3\pi }\sin\;\left(2\pi 3f_{s}t\right)+\frac{4}{5\pi }\sin\;\left(2\pi 5f_{s}t\right)+\ldots$$

and the filtered sensor signal is given by:

(5) $$u_{F}\left(t\right)=A\sin\;\left(2\pi f_{s}t+\varphi \right)$$

where for simplicity, the amplitude is denoted as *A*, where A∝E, and *φ* is the phase difference between the optoelectronic sensor signal and the sensor signal, which is either 0° or 180°, depending on the field polarity.

**Figure 3:**
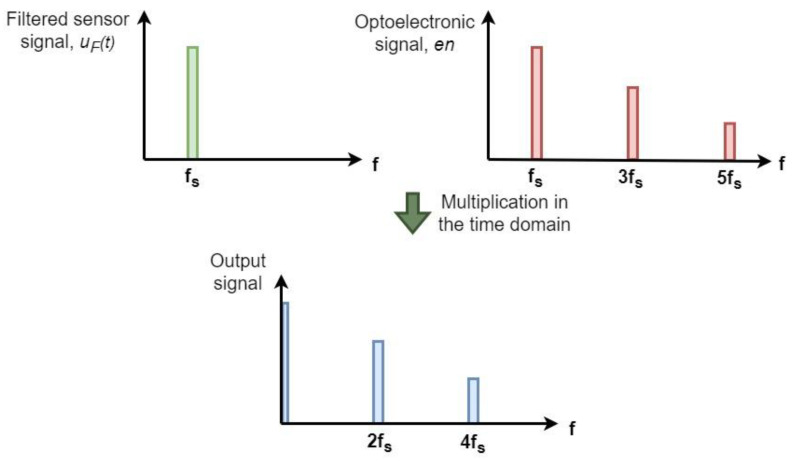
Multiplication in the time domain—spectrum analysis.

The multiplication of *u_F_(t)* by each term of *en,* would result in two frequency components at the sum and difference frequencies for each term, as shown in Figure 3.

Therefore, the signal at point D would theoretically contain frequency components at the even harmonics of the fundamental frequency, as well a DC term, which results from the multiplication of the sensor signal by the first odd harmonic of the square wave:

(6) $$\frac{4}{\pi }\sin2\pi f_{s}t\times A\sin\;\left(2\pi f_{s}t+\varphi \right)=\frac{4A}{2\pi }\cos\varphi -\frac{4A}{2\pi }\cos\;\left(2\pi 2f_{s}t+\varphi \right)$$

The first term of the product in (6) is the DC term, and it is proportional to both the amplitude *A* and the phase difference *φ*. Since *φ* is either 0° or 180°, for positive and negative polarity of the electric field, respectively, the DC term acquires positive or negative values, accordingly. Thereby, using a low pass filter to suppress the higher even harmonics would result in a DC voltage that reveals the magnitude and the polarity of the electric field.

**Figure 4:**
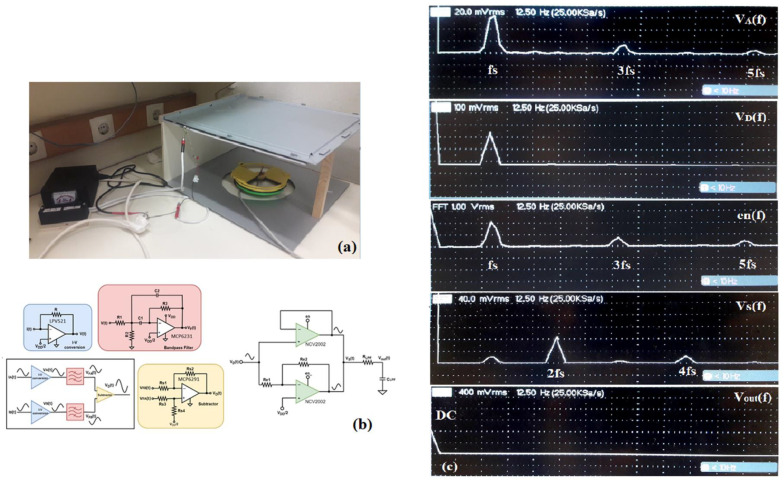
(**a**) 3D-printed field mill and calibration setup (**b**) Schematic of the discrete implementation of interface circuitry (**c**) FFT of signals.

Figure 4a,b depict the prototype 3D-printed field mill sensor used in [11] and the calibration setup as well as the sensor interface. The design logic of the interface circuitry was similar, i.e., the preamplification of the signal, noise filtering by a narrow bandpass filter, and synchronous demodulation, but a multiple feedback narrow bandpass filter configuration was employed in this case. Figure 4c includes an FFT analysis of the raw signal, V_A_(t); the filtered signal, V_D_(t); the optoelectronic sensor, en(t); the synchronously demodulated signal, V_S_(t); and the low pass filtered output signal, V_out_(t).

A closer examination on the noise filtering behavior would lead to the conclusion that after the bandpass filter, the desired signal along with the flicker and thermal noise components at the passband is obtained. Since the frequency of the desired signal is limited by the sensor design to be at the frequency range where flicker noise is the dominant source of noise, the preamplification stage should be designed to have minimum noise contribution and, more specifically, minimum flicker noise contribution. This was the object of study of a previous work [11], and an improved amplifier design more suitable for the application, which adopted the low-noise and low-power design techniques utilized in [15,16], was used in this work (see Section 4). 

Table 1 includes some design parameters of the field mill sensor design studied in this work. The motor frequency of 75 Hz was selected as a balance between the proper movement of the rotating vanes (stable movement under 100 Hz [14]) and the derived signal frequency, which should be as high as possible for lower flicker noise contribution of the preamplifier since flicker noise is inversely proportional to the frequency. The number of vanes was selected based on the study conducted in [8], as a balance between the induced current and the effect of edge effect which increases with the number of vanes.

## 3. Integrated Filter Design Theory and Techniques

Fully integrated filter design techniques specifically aimed to optimize the filter design at the low frequency domain in terms of output noise, power consumption, dynamic range, total harmonic distortion (THD), etc. were studied, and the final design is used in the integrated sensor interface. Four OTA-based cascaded topologies with passive element replacement and one implementation using the leapfrog technique in an RLC filter topology were designed and compared for integrability, silicon area, performance degradation at process corners, temperature variations, supply voltage variations, etc.

### 3.1. Higher Order Filters Using Cascaded Topologies

**Figure 5:**
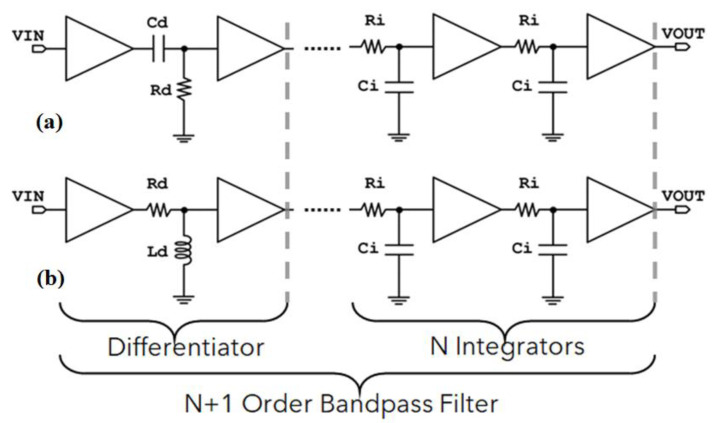
(n + 1)th order bandpass filter (**a**) CR high-pass filter implementation, (**b**) RL high-pass filter implementation.

Figure 5 depicts the *CR-RC^n^* and the *RL-RC^n^* configuration of the (n + 1) order bandpass filter, which consists of an CR or RL differentiator and *n* integrators [17]. Cascading 1st order filters results in higher order filter topologies. The lower frequency bound of this topology is given by:

(7) $$f_{c\left(Lowpass\right)}=\frac{1}{2\pi R_{i}C_{i}}\;$$

whereas the upper frequency bound for the *CR-RC^n^* and the *RL-RC^n^* is given by:

(8) $$f_{c\left(Highpass\right)}=\frac{1}{2\pi R_{d}C_{d}}=\frac{R_{d}}{2\pi L_{d}}$$

The transfer function of the *CR-RC^n^* bandpass filter, if *τ_d_* is the time constant of the differentiator, if *τ_i_* is the time constant of the integrators, and if *A* is the dc gain of the integrators, is given by [18]:

(9) $$H\left(s\right)=\left(\frac{s\tau_{d}}{1+s\tau_{d}}\right){\left(\frac{A}{1+s\tau_{i}}\right)}^{n}$$

### 3.2. Cascaded Topologies with OTA-Based Passive Element Replacement

**Figure 6:**
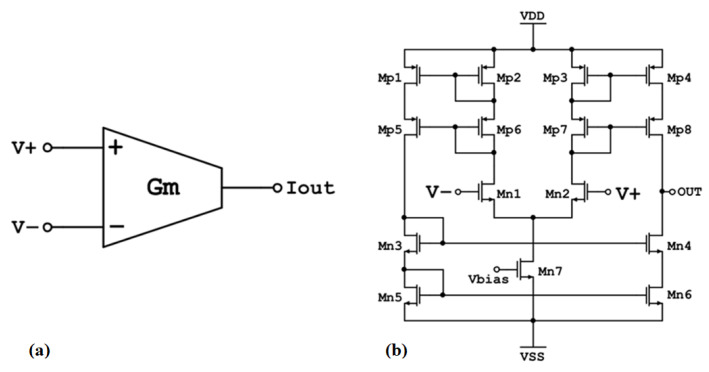
(**a**) Symbol of Operational Transconductance Amplifier (OTA) (**b**) Schematic of CMOS OTA.

OTA-based simulator configurations can replace passive components that are challenging to integrate due to their high values [19]. The OTA circuit (Figure 6) has a differential voltage input and an output current that is a linear function of this voltage:

(10) $$I_{out}=G_{m}\left(V^{+}-V^{-}\right)$$

Additionally, the OTA circuit’ s transconductance, *G_m_*, can be adjusted through the bias current of the amplifier ($G_{m}\propto \;\sqrt{I_{bias}}$). The OTA symbol as well as its schematic design are depicted in Figure 6a,b, respectively.

**Figure 7:**
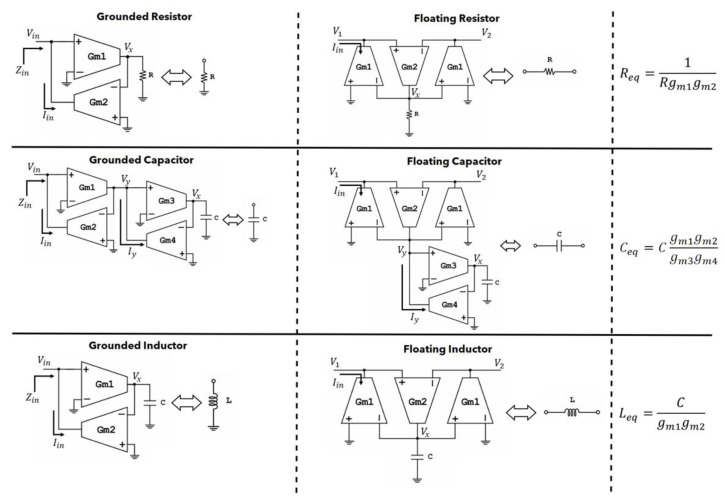
Passive component replacement using OTAs.

In Figure 7, the passive element replacement OTA-based topologies with their respective equivalence formulas are depicted [20,21].

**Figure 8:**
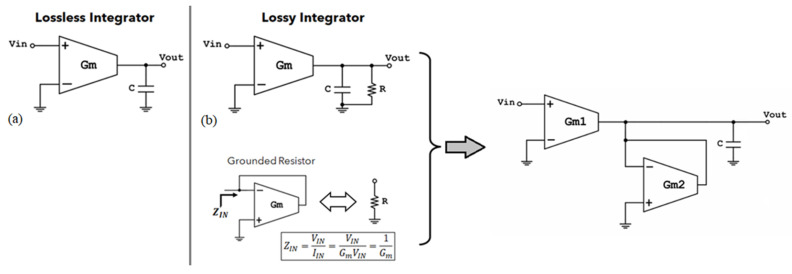
(**a**) Lossless integrator, (**b**) Lossy integrator with element replacement.

Figure 8 depicts two OTA-based integrator blocks for the LP part of the cascaded filter design, i.e., the lossless and the lossy integrator [20,22]. The transfer function of the lossless integrator is given by:

(11) $$H\left(s\right)=\frac{G_{m}}{sC}$$

and the transfer function of the lossy integrator is given by:

(12) $$H\left(s\right)=\frac{G_{m}R}{1+sRC}$$

Replacing the resistor of the lossy integrator by an OTA-based equivalent grounded resistor (Figure 8b) results in a lossy integrator with an adjustable gain, *K*:

(13) $$H\left(s\right)=\frac{K}{1+\tau s}$$

where $K=G_{m1}/G_{m2}$, and $\tau =C/G_{m2}.$

**Figure 9:**
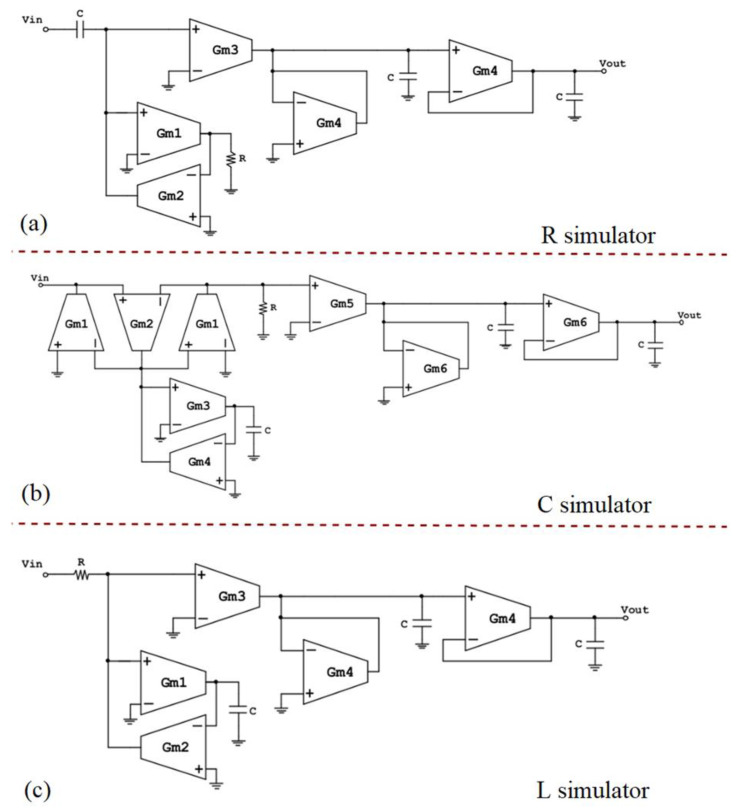
OTA-based passive element replacement bandpass filters—cascaded topologies (**a**) R simulator, (**b**) C simulator, (**c**) L simulator.

In Figure 9, the three different OTA-based passive element replacement BPF implementations are depicted. These configurations are based on the (n + 1)th order bandpass filter of Figure 5, which consists of a differentiator (CR or RL) and n integrators. More specifically, Figure 9a depicts the design of the bandpass filter, in which the R of the CR differentiator is replaced by its OTA-based equivalent. In Figure 9b, the C of the CR differentiator is replaced by its OTA-based equivalent, whereas in Figure 9c, the L of the RL differentiator is replaced by its OTA-based equivalent. In all three implementations of Figure 9, the integrator part consists of two integrators, i.e., one lossy integrator with R replacement (as presented in Figure 8b) and one lossless integrator, whose inverting input is connected to its output. From this configuration, the transfer function in (11) becomes:

(14) $$H\left(s\right)=\frac{1}{1+\tau s}$$

where $\tau =\frac{C}{G_{m}}$, which results in:

(15) $$f_{c}=\frac{1}{2\pi \tau }=\frac{G_{m}}{2\pi C}$$

Thereby, the bandwidth of the low-pass filter can be easily adjusted by the OTA’s bias current, since $\left(G_{m}\propto \;\sqrt{I_{bias}}\;\right)$ and should introduce the same cutoff frequency that the lossy integrator introduces.

The purpose of the lossy integrator with R replacement is to introduce a controlled output gain—since a lossless integrator does not provide gain—which is a desirable feature in the bandpass filter design and which can be adjusted from (13).

Table 2 includes the transfer function of each filter implementation of Figure 9, as well as the expressions for the upper frequency bound, *f_c(highpass)_*, and the lower frequency bound, *f_c(lowpass)_*, and the midband gain.

### 3.3. Bandpass Filter Using Leapfrog Technique

**Figure 10:**
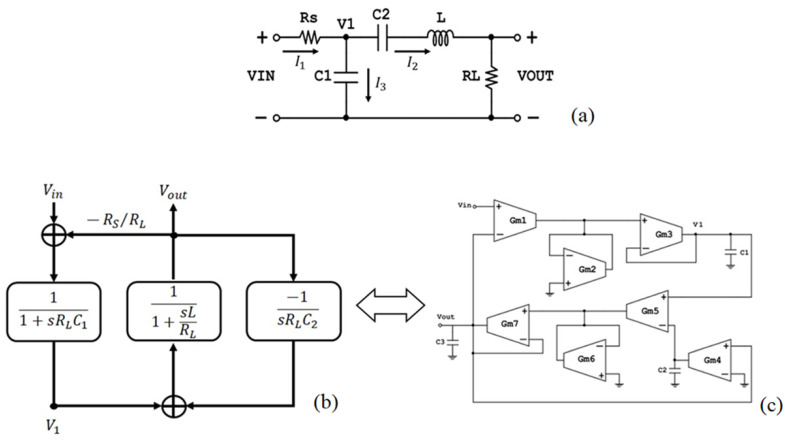
(**a**) OTA-based 3rd Order Bandpass Filter implementation, (**b**) Signal flow, and (**c**) Leapfrog technique (RLC two port topology).

In Figure 10a the passive RLC two port circuit topology of a third-order bandpass filter is depicted [12,23]. 

Application of Kirchhoff’s Current Law (KCL) on node *V*_1_ (Figure 10a) results in:

(16) $$I_{1}=\frac{V_{in}-V_{1}}{R_{S}},\;I_{2}=\frac{V_{out}}{R_{L}}\;and\;I_{3}=sC_{1}V_{1}$$

and since $I_{1}=I_{2}+I_{3}$:

(17) $$V_{1}=\frac{1}{1+sR_{L}C_{1}}\left(V_{in}-\frac{R_{S}}{R_{L}}V_{out}\right)$$

From $I_{2}=\frac{V_{out}}{R_{L}}=\frac{V_{1}-V_{out}}{\frac{1}{sC_{2}}+sL}$:

(18) $$V_{out}=\frac{\left(V_{1}-\frac{V_{out}}{sR_{L}C_{2}}\right)}{1+\frac{sL}{R_{L}}}$$

From (17) and (18), the signal flow shown in Figure 10b is derived. The leapfrog filter by element replacement is depicted in Figure 10c.

### 3.4. Filters Comparison

**Figure 11:**
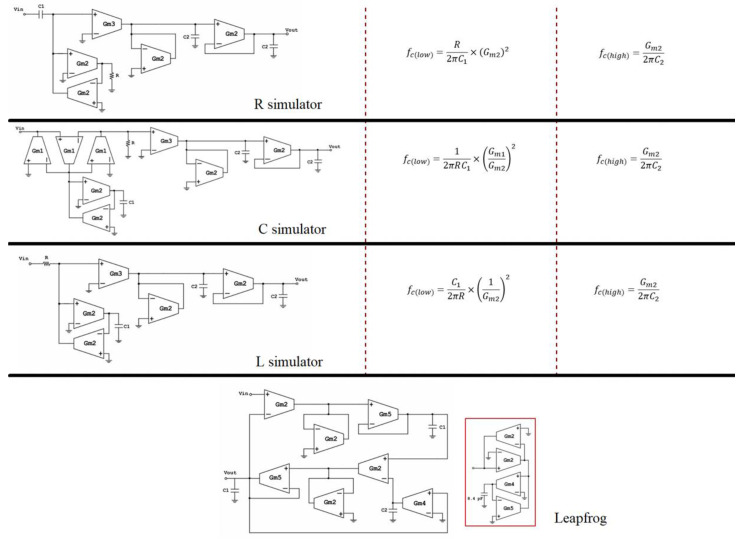
Final versions of the filter designs and their respective updated high-pass and low-pass cutoff frequency expressions.

Figure 11 depicts the finalized filter designs along with the respective, accordingly modified, high and low cutoff frequency expressions.

Table 3 includes the passive components and the transconductances of each filter implementation. All these values were adjusted to achieve the desired passband of 20 Hz–10 kHz in the bandpass filters, as well as the desired midband gain of 12 dB. The value of capacitor C2 of the leapfrog design cannot be integrated; therefore, the corresponding OTA-based simulator is depicted in the red frame of Figure 11.

Table 4 includes the transistor dimensions of each OTA design used for the filters’ design. These dimensions were carefully selected to achieve the desired transconductance values, the low noise requirements, and the low power demands of the application.

Table 5 includes the transconductance values of each OTA at process corners and the mean value of each transconductance derived from Monte Carlo analysis. The derived mean values from the Monte Carlo analysis appear in Table 5. This analysis takes into consideration the device mismatches, and it is more conservative compared to corner analysis; thereby, the mean values derived from the Monte Carlo are expectedly closer to the nominal values for each transconductance.

The Fast–Fast corner case sets all the devices models to a worst-power (WP) condition. In this condition, both NMOS and PMOS transistors are considered fast, and all passive component values (R and C) are smaller than expected (higher variations in resistors than capacitors). The Slow–Slow corner case sets all the devices models to a worst-speed (WS) condition. In this condition, both NMOS and PMOS transistors are considered slow, and all passive component values are larger than expected. The Typical–Typical corner uses the nominal device values for all transistors and passive components of the circuit under test without any deviation from the expected values.

**Figure 12:**
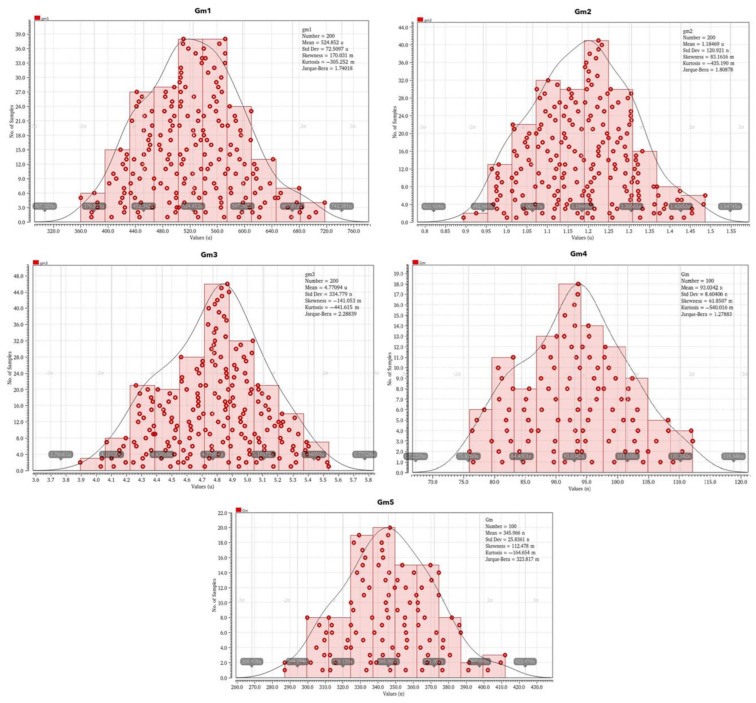
Monte Carlo analysis for each OTA transconductance.

Figure 12 depicts the derived diagrams of the Monte Carlo analysis of each OTA transconductance value G_m1_–G_m5_. Monte Carlo analysis is a process/mismatch statistical-oriented analysis in which a critical performance metric of a circuit can be tested along process corners and global/local mismatch variations. Using a large number of samples, Monte Carlo device models, and a technology-dependent standard deviation (sigma), the impact of both process corners and mismatch variations along the design can be simulated. The Monte Carlo results are provided using a histogram approach for better visualization. The X-axis depicts the calculated value of the desired performance metric, i.e., gain, cut-off frequency, transconductance, etc., whereas the Y-axis illustrates the number of Monte Carlo samples for the desired metric calculation, i.e., the probability of calculating a specific X-axis value. When all the Monte Carlo iterations are finished, a Gaussian-like probability distribution graph is derived, and the calculated performance metric values with the highest and the lowest probabilities can be extracted. Regarding Figure 12, the X-axis represents the calculated transconductance G_m_ (μA/V) of each OTA design which is a crucial performance metric in the filter design based in OTAs, in terms of the cut-off frequencies and the desired gain. The Y-axis represents the number of times that the X-axis transconductance value was calculated. Thus, the G_m_ value of each OTA design with the highest probability can be derived and compared to the typical value calculated in the design process (typical corner strategy).

Table 6 includes the power consumption value of each filter implementation at typical model (TM), worst power (WP), and worst speed (WS) corners, as well as the mean value derived from the Monte Carlo analysis. A corner strategy of the Typical–Typical (TM), Fast–Fast (WP), and Slow–Slow (WS) corners is presented. At the Fast–Fast corner (FF), resistors have a smaller value than the expected, and both NMOS and PMOS transistors have higher mobility. Thus, this corner is considered as the worst-power (WP) corner. Using this process corner case, the overall power of the designed circuit is significantly higher than initially calculated. This power consumption value is the highest power that the circuit can consume due to process/mismatch variations. On the other hand, at the Slow–Slow corner (SS), resistors have larger value than the expected, and both NMOS and PMOS transistors have lower mobility. Thus, this corner is considered as the worst-speed (WS) corner while the power consumption value, at this corner, is the lowest power that the circuit can consume. By performing a Monte Carlo analysis and monitoring the power consumption, the power consumption with the highest probability can be derived.

The C simulator configuration has the highest power consumption; however, it is comparatively low for the requirements of this application, where the motor consumes a few hundreds of milliwatts to a few Watts.

Table 7 includes the output noise value of each filter implementation at TM, WP, and WS corners. The same corner strategy as in Table 6 was employed. The output rms noise of the filter, at each specific process corner, was calculated by integrating the noise PSD curve of the circuit (integrated noise) using a frequency range derived from the filter’s bandwidth (20 Hz–10 kHz). There is no need of addressing the output noise of the filter outside of its bandwidth because it will be suppressed by the filter anyway. A significant filter metric that indicates the minimum input signal that can be processed by the filter is the Signal-to-Noise ratio (SNR). The SNR can be calculated using the formula below:

(19) $$SNR\;\left(dB\right)=20\times \log\left(\frac{V_{o\left(rms\right)}}{V_{noise\left(rms\right)}}\right)=20\times \log\left(\frac{V_{in_{peak}}\times A_{v}}{\sqrt{2}\times V_{noise\left(rms\right)}}\right)$$

where Av is the gain of the filter, and the V_in_peak_ is the input signal amplitude. When the SNR = 0 dB, the input signal is equal to the noise floor of the circuit which indicates the minimum distinguishable input signal of the filter. Techniques for low frequency operation OTA designs combined with a low corner frequency [24,25] were implemented. From this table, it is observed that the R simulator configuration has considerably higher output noise compared to the other three topologies.

**Figure 13:**
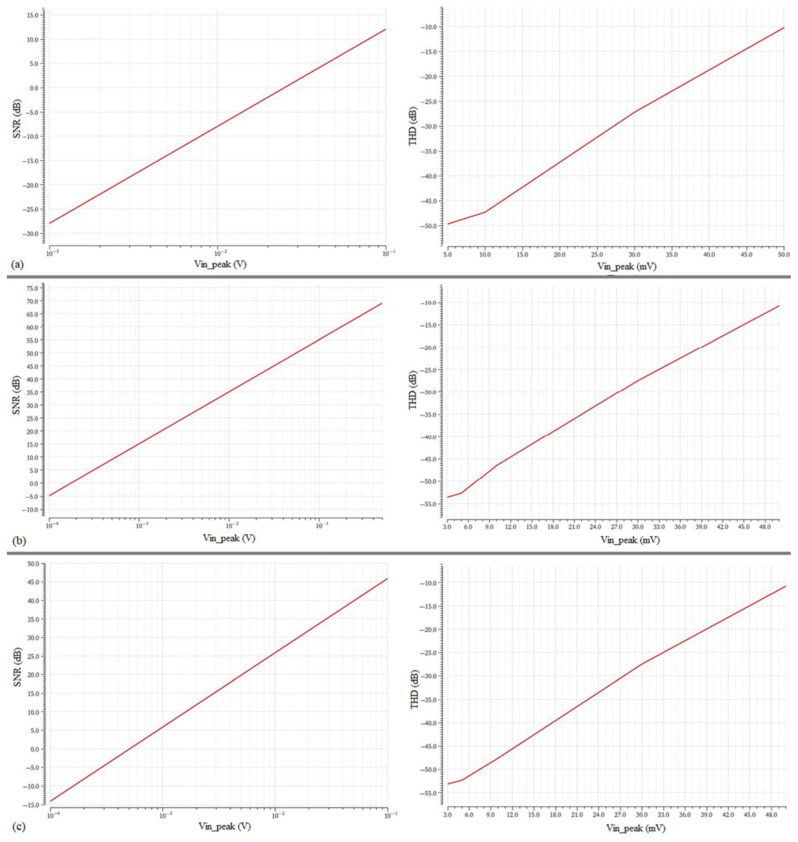
Signal-to-Noise Ratio (SNR) vs. V_in_peak_ and Total Hamonic Distortion vs. V_in_peak_ for the (**a**) R replacement, (**b**) L replacement, and (**c**) C replacement cascaded bandpass filter configuration.

In Figure 13, the THD and SNR plots versus the amplitude of the input signal, V_in_peak_, for the cascaded filter configurations are depicted. 

**Figure 14:**
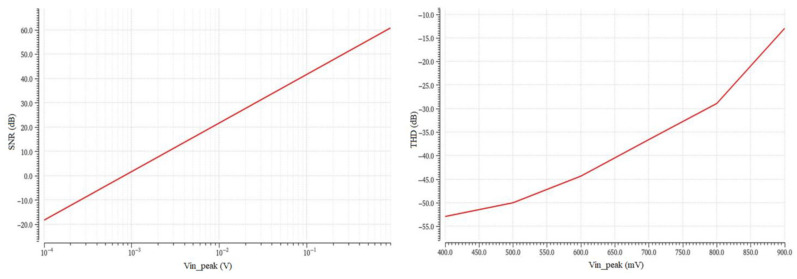
Signal-to-Noise Ratio (SNR) vs. V_in_peak_ and Total Hamonic Distortion vs. V_in_peak_ for the leapfrog topology.

In Figure 14, the THD and SNR plots versus V_in_peak_ for the leapfrog technique are depicted. From the diagrams in Figure 13 and Figure 14, the values that appear on Table 8 are extracted.

Table 8 includes TM, WP, and WS corner analysis of the midband gain, as well as the V_in_peak_ values for THD = −40 dB and SNR = 0 dB, and the derived dynamic range (DR) for each filter implementation, according to:

(20) $$DR=20\log\frac{V_{\mathrm{in}\_\;\mathrm{peak}\left(@THD=-40\;\mathrm{dB}\right)}}{V_{in\_peak_{(@SNR}=0\;\mathrm{dB})}}$$

The value of V_in___peak_ at THD = −40 dB indicates that each filter implementation can accommodate signals with an amplitude of up to this voltage, after which the THD becomes greater than 1%. The value of V_in___peak_ at SNR = 0 dB indicates the amplitude of the minimum discernible signal; input signals should be higher than this signal to be readable. The DR value, which is derived from (20), indicates the ratio between the strongest non distorted signal and the minimum readable signal. The V_in___peak_ at SNR = 0 dB of the R simulator is considerably higher than its V_in___peak_ at THD = −40 dB; therefore, it cannot work properly. From Table 8 it is observed that the leapfrog topology cannot achieve the desirable gain of 12 dB.

Table 9 includes the Process, Voltage and Temperature (PVT) corners of the high pass frequency, f_c(Highpass)_, the low pass frequency, f_c(Lowpass)_, and the gain for the cascaded filter topologies. Table 10 includes the results from the same analysis for the leapfrog filter topology. PVT corner analysis is conducted to test the integrated circuit in extreme and varying conditions of process variations, voltage, and temperature. Process corners SS, TT, and FF refer to the Slow–Slow, Typical–Typical, and Fast–Fast corners which were previously described for Table 6. In voltage corner analysis, the bias voltage Vbias of the OTA designs is given values that deviate from its nominal value (−1.1 V), whereas in temperature corners, the temperature is set at ± 40 °C and at 0 °C to test the behavior of the passband frequencies under extreme conditions.

The initial filter designs were tested for an indicative passband of 20 Hz–10 kHz, where f_c(Highpass)_ = 20 Hz and f_c(Lowpass)_ = 10 kHz. At process corners, the R simulator and the L simulator showed a deviation from the nominal high-pass frequency of 65% and 66%, respectively, whereas the C simulator showed a deviation of 2%, which is significantly lower. Leapfrog showed a deviation of 45%. The R, L, and C simulators showed a deviation of 48% from the nominal low-pass frequency, whereas the leapfrog topology showed a deviation of 46%.

A comparison of all the above filter configurations shows that the cascaded topology employing C replacement and the leapfrog filter design have the best performance in terms of cutoff frequency behavior at process corners, where C replacement shows significantly lower deviation of the high-pass frequency from the leapfrog topology. This is due to the transconductance ratio-dependence of the capacitance C_eq_ of the C replacement topology:

(21) $$f_{c\left(\mathrm{Highpass}\right)}=\frac{1}{2\pi RC_{1}}\times {\left(\frac{G_{m1}}{G_{m2}}\right)}^{2}$$

These configurations are additionally characterized by low output noise, while also providing a high dynamic range. The R replacement topology is not selected since it cannot work properly (DR < 0) due to the high noise floor. The behavior of the L replacement topology at process corners, where its high-pass frequency varies significantly, makes it unsuitable for the specific application, where the passband should be mostly stable. The significant variation of the high-pass frequency in this topology in process corners is due to the non-ratio-dependence of the inductance L_eq_:

(22) $$f_{c\left(\mathrm{Highpass}\right)}=\frac{C_{1}}{2\pi R}\times {\left(\frac{1}{G_{m2}}\right)}^{2}$$

The low values of THD in the cascaded bandpass filters configurations with R, L, and C replacement are caused due to the employment of higher effective transconductances, which result in a limited linearity range of the OTA. Since highly linear OTAs result in reduced THD, the leapfrog topology outperforms the cascaded configurations in terms of this parameter. The C replacement topology outperforms the leapfrog topology since the latter cannot provide the desired gain. Therefore, as the final filter design the C replacement was selected and redesigned to satisfy the specifications of the field mill sensor application.

### 3.5. Narrow Bandpass Filter

**Figure 15:**
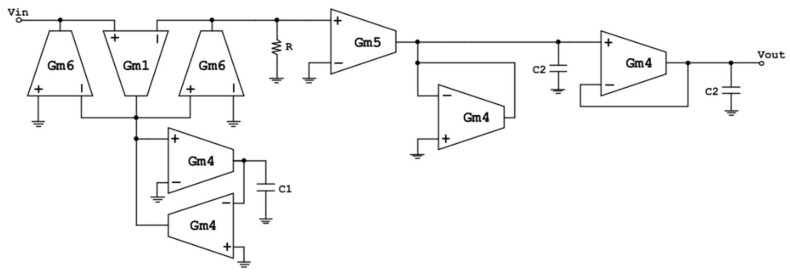
OTA-based 3rd Order Narrowband Bandpass Filter circuit design for low frequency applications.

Combining the *CR-RC^n^* bandpass filter implementation using C replacement for the capacitor of the high-pass filter and a combination of a lossy and a lossless integrator for the realization of the low pass filter, the final schematic of the optimized third-order bandpass filter was designed, which appears in Figure 15. The filter was redesigned and optimized in terms of power consumption, output noise, and passband bandwidth to better fit the field mill sensor specifications.

The specifications of this filter (Table 11) were determined by the parameters of the field mill sensor device, such as the motor rotation frequency, the number of vanes of the shielding and sensing electrodes, and the known noise frequency components (slow linear trends, odd harmonics, etc.). The passband of the final BPF designed was narrowed down to exclude the maximum possible noise components (20 Hz–1 kHz), and a gain of 4 (12 dB) was considered adequate for the interface design.

The main parameter values of the filter of Figure 15 are presented in Table 12. This table includes all the transconductances of the OTA designs used for the final filter design, the values of the passive elements, and the values of the symmetrical supply voltages.

More specifically, the transistor values of each OTA design for the filter implementation of Figure 15 appear in Table 13. The transistor names correspond to the transistors of the OTA design of Figure 6b, since the same OTA configuration was employed for all the OTA circuits used in the filter design. The transistor sizing was carefully selected and optimized to satisfy the demands of both low area physical design of the filter and to accommodate several design parameters requirements, such as low noise and low power consumption as well as specific transconductance values.

Table 14 includes the process corner analysis results for each transconductance value of the final filter design, as well as a Monte Carlo analysis. A simple corner strategy, Typical–Typical, Fast–Fast, and Slow–Slow corners, was used as to derive the transconductances values of the OTAs, illustrated in Table 14, at those three corners. Moreover, a Monte Carlo analysis was performed to calculate the highest probability transconductance value of each OTA when both process and mismatch variations are considered. The Monte Carlo results of the OTA transconductances (provided as mean transconductance values in Table 14) have insignificant deviation from the value calculated using the typical corner case. Thus, the most probable transconductance value of each OTA, when process/mismatch variations are considered, is almost equal to the calculated value in the typical corner case.

In Table 15, the power consumption of the filter using a corner strategy (same as the one employed in Table 6) of the Typical–Typical (Typical Model, TM), Fast–Fast (Worst Power, WP), and Slow–Slow (Worst Speed, WS) corners is presented. By performing a Monte Carlo analysis and monitoring the power consumption, the power consumption with the highest probability can be derived.

The power consumption of the filter, even in extreme worst power (WP) conditions, is very low compared to the power consumption of the motors in field mill applications, which typically ranges from a few hundreds of milliwatts to a few Watts.

Table 16 includes the same corner strategy analysis results concerning the output rms noise of the filter as in Table 7, which does not significantly vary and acquires its lower value when the power consumption is higher (WP corner). The output rms noise of the filter, at each specific process corner, was calculated by integrating the noise PSD curve of the circuit (integrated noise) using a frequency range derived from the filter’s bandwidth (20 Hz–1 kHz). 

**Figure 16:**
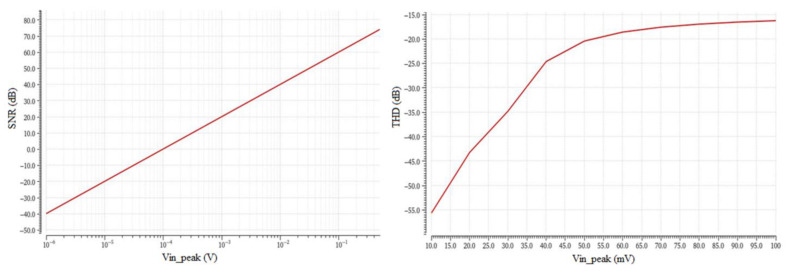
Signal-to-Noise Ratio (SNR) vs. V_in_peak_ and Total Harmonic Distortion (THD) vs. Vin_peak.

Figure 16 depicts the SNR and the THD versus V_in_peak_. The value of V_in_peak_ at THD = −40 dB indicates the maximum input amplitude voltage that can be processed by the filter with a total harmonic distortion of 1%. Thus, for signals with amplitude greater than 23.8 mV (peak amplitude), the THD component becomes greater than 1% (or greater than −40 dB). This THD condition sets the upper limit of the input signal that the filter can accommodate. The lower limit of the input signal can be derived from the value of V_in_peak_ at SNR = 0 dB which indicates the minimum discernible signal of a peak voltage of 97.9 μV. Using these two input signal limits, the dynamic range (DR) of the designed filter can be easily calculated.

In Table 17, the results from the SNR and the THD analyses of Figure 16 are presented more analytically. Again, a process corner analysis using the TM, WP, and WS corner strategy was performed to extract the midband gain deviation across those three corners. Furthermore, the calculated values for SNR = 0 dB and THD = −40 dB (or 1%) of the input amplitude voltage V_in_peak_ were also presented. These two V_in___peak_ values were calculated using the typical corner case (TM) and represent the lower and upper input voltage limits that the filter can process. By using these two V_in___peak_ values (amplitude peak voltages), the dynamic range of the filter was also calculated.

**Figure 17:**
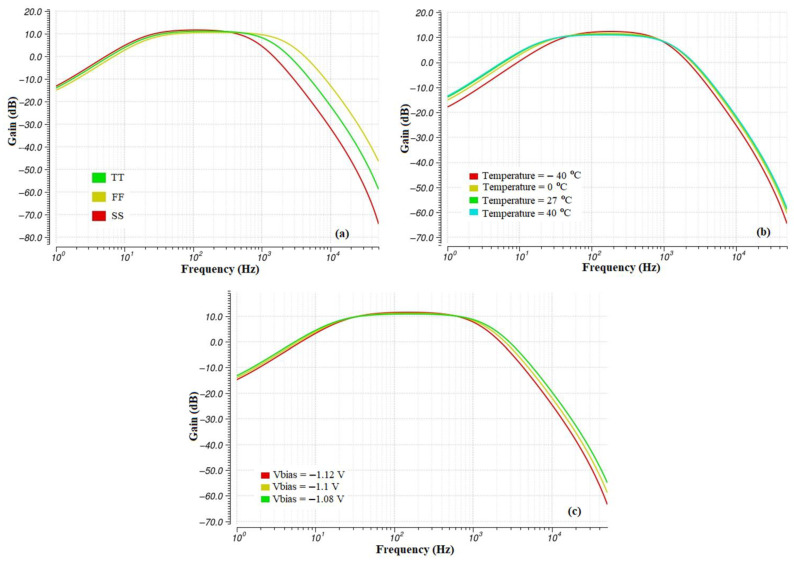
PVT corner—AC analysis (**a**) Process corners, (**b**) Temperature corners, and (**c**) Voltage corners.

Finally, Figure 17 shows the AC analysis of the filter in PVT corners. The bandpass behavior of the filter is apparent, as well as the variations of the high-pass and low-pass frequencies for each type of corner analysis. The most significant variation of the high-pass frequency is observed at the temperature corners, whereas the most significant variation of the low-pass frequency is observed at the process corners. The midband gain remains stable at all PVT corners.

Table 18 includes the results of the PVT corners of Figure 17 for the high-pass frequency, f_c(Highpass)_, for the low-pass frequency, f_c(Lowpass)_, and the gain. The optimized narrow bandpass filter shows a deviation of 6.8% from the nominal high-pass frequency and a 48% deviation from the nominal low-pass frequency, while showing significantly smaller variations at voltage and temperature corners.

In Table 9, the cutoff frequency values of the cascaded filter topologies are satisfactory, in that the derived passband at every corner analysis includes and amplifies the frequency of the expected sensor signal, which is 450 Hz. However, the results in Table 18, where simulations were conducted for the optimized design with the narrower passband, show that in all PVT corners the passband includes and amplifies the useful signal of 450 Hz, while it additionally excludes a higher number of unwanted noise components outside the passband range.

The midband gain of the optimized filter design (Table 18) is a little lower than the initial design’s gain at PVT corners (Table 9); however, this was alleviated by the extra amplification stage after the filter which was employed to counteract the limited dynamic range of the filter—as will be described in Section 4.

## 4. Simulation Results

**Figure 18:**
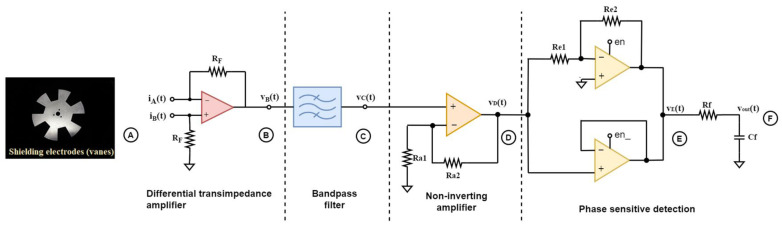
Final sensor interface design.

To demonstrate the effect of the bandpass filter in the noise rejection and the overall sensitivity enhancement, transient noise simulations as well as spectrum analysis at each stage were conducted. The finalized design of the analog readout front-end is depicted in Figure 18. The bandpass filter used was the optimized version of the C replacement cascaded topology. A non-inverting amplifier is added after the bandpass filter to further amplify the signal, as to improve the overall sensitivity, as well as to act as a buffer between the filter and the phase-sensitive detection stage. The non-inverting amplifier’s gain, A, is given by:

(23) $$A=1+\frac{Ra2}{Ra1}=21$$

and was selected as to exploit the better part of the voltage supply range (VDD = 1.8 V, VSS = −1.8 V).

Table 19 includes the values of all the passive components used for the design of Figure 16.

**Figure 19:**
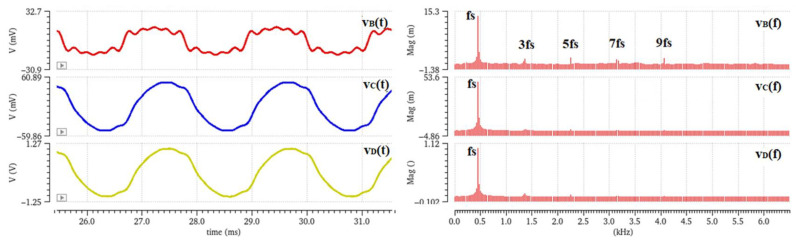
Transient and frequency analysis of the signals before/after filtering and after amplification—odd harmonics of the fundamental frequency are added to the raw sensor signal.

All simulations presented below are post-layout simulations, i.e., the effect of the extracted parasitics in simulations is included. In Figure 19 the transient and frequency analysis of the signal at each stage is presented. The first three signals, v_B_(t)/v_B_(f), v_C_(t)/v_C_(f), and v_D_(t)/v_D_(f), demonstrate the effect that the filtering has on the noise introduced by the odd harmonics—which in the actual application are caused by the sensing electrodes’ shape. More specifically, during simulations, noise signals were added to the raw sensor signal—which is realized by a current source—at the odd harmonics of the fundamental signal frequency. These signals represent the noise that is introduced by the sector-shaped sensing electrodes, which significantly distorts the already weak current signal, and they can be observed at the output voltage signal, v_B_(t), after preamplification (Figure 19). The narrow bandpass filter excludes these harmonics from the sensor signal, and only the frequency component of interest, v_C_(t), is synchronously demodulated. Before demodulation, the signal is further amplified, and signal v_D_(t) is obtained. 

**Figure 20:**
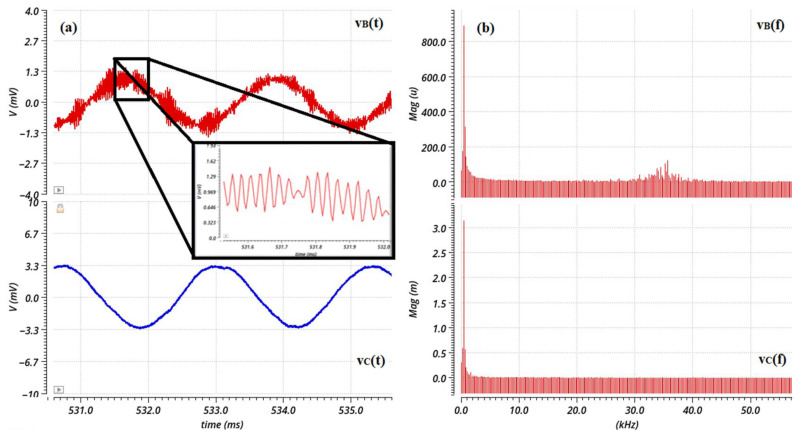
(**a**) Transient noise analysis and (**b**) spectrum analysis of the signal before and after filtering.

A transient noise analysis reveals the effect of filtering on the jitter noise before and after filtering. Transient noise analysis includes the noise introduced by the devices (flicker, thermal, and shot). This simulation includes only the noise introduced by the circuitry, i.e., mainly the preamplification stage. Figure 20a show the transient noise analysis results, whereas Figure 20b shows the respective spectrum analysis of each signal.

**Figure 21:**
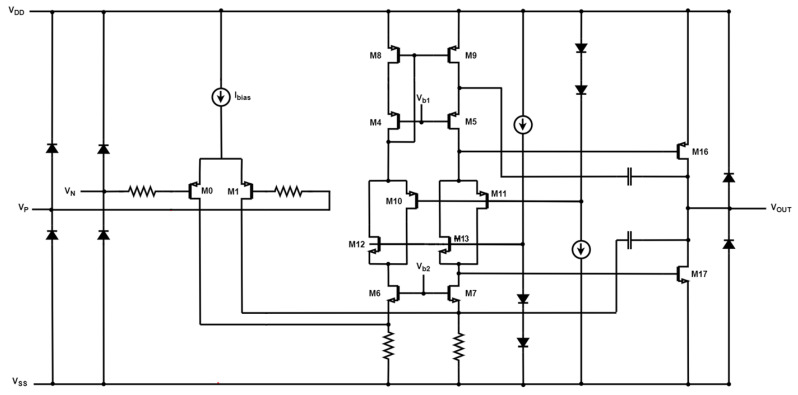
Op-amp schematic for the preamplification stage.

Taking into consideration the superposition principle, the spectrum analyses in Figure 19 and Figure 20 reveal that at point B in Figure 18, the signal, v_B_(t), is affected by the noise introduced by the preamplifier as well as the harmonics caused by the sector-shaped sensing electrodes. After the narrow bandpass filter, the signal, v_C_(t), is mostly exempted from the (odd) harmonics and the noise components outside of the passband. At this point, only the desired signal, along with the flicker and thermal noise that belongs inside the limits set by the low cutoff frequency and the high cutoff frequency of the filter, is obtained and forwarded at the phase sensitive detection stage. Therefore, it is important that the preamplifier introduces minimum flicker noise since the signal belongs in the low frequency domain. The current signal that is measured from the field mill sensing electrodes is a low-level signal, i.e., in the pA-nA range. Therefore, the op-amp employed for the preamplification stage should have very low input bias currents, as well as low input current noise. These requirements were the focal point of [11], where the op-amp design was optimized to combine low input current and voltage noise, as well as low bias currents, by employing noise minimization techniques and applying them to the sizing process of the MOSFET devices of the op-amp. This class AB amplifier (Figure 21) consists of a PMOS differential pair. The input differential pair transistors, as well as the transistors that compose the summing circuit of the amplifier, are the main noise contributors of the amplifier design. Since the flicker noise is inversely proportional to the WL area of the transistor device, the W and L dimensions of these transistors were increased to decrease the flicker noise [15,16].

Table 20 includes the voltage and current noise of the op-amp that was employed for the preamplifier design. The input-referred voltage and current spot noise of the op-amp, which was simulated at the frequency of the expected sensing signal (450 Hz), is comparable to the noise of discrete IC op-amps. It was achieved by employing noise optimization techniques as thoroughly described and analyzed in a previous work [11].

Table 21 includes the power consumption of the main building blocks as well as the total power consumption of the sensor interface. The main energy demands come from the preamplifier and the filter. The total power consumption of the interface circuitry is very low, and compared to the power demands of the motor, which is in the order of hundreds milliwatts to a few Watts, it can be considered negligible. 

In this application, the motor consumption is approximately 480 mW (@ 3 V), which, even in an intermittent style of operation such as the one suggested in [11], where the motor would not operate continuously, is still much higher that the power consumption of the filter (404.3 μW) and the sensor interface (1.13 mW) for that matter.

**Figure 22:**
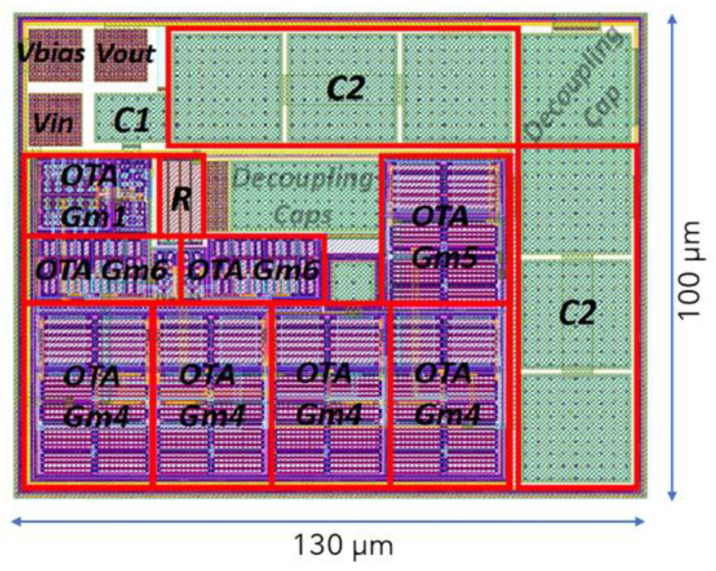
Layout of the optimized bandpass filter.

In Figure 22, the physical design (layout) of the bandpass filter is depicted. The OTA designs and the passive elements that correspond to the transconductance and the resistor/capacitor values in Table 12 can be seen. The OTA design transistor values were presented in Table 13.

In integrated circuit design, the available area for the System-On-Chip is limited, while also the cost of manufacturing rises significantly with the chip size. Therefore, it is important for the designs to be as compact as possible and occupy as little area as possible. 

As it is mentioned in the Introduction section, the main challenge during integrated filter design, specifically in the low frequency domain (such as in the case of this application), is the integration of the passive elements, which for the conventional filter configurations should have high values and be bulky. This issue was addressed in this work, where the passive elements were replaced by OTA-based equivalent circuits. The final area of the physical design of the filter is 130 μm × 100 μm, which is remarkably small.

**Figure 23:**
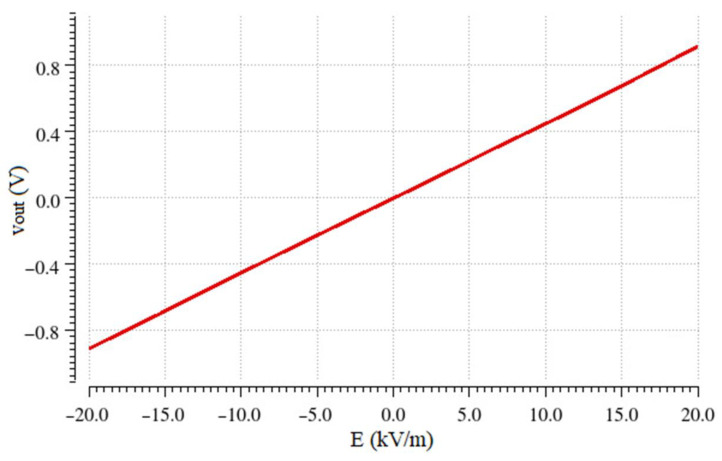
Output voltage vs. electric field (Linear equation: V_out_ = 5 × 10^−7^ E—0.0039 V, R^2^ = 1).

Figure 23 depicts the output voltage value after low pass filtering versus the theoretical electric field. For electric field with positive polarity, the output voltage is positive, whereas for electric fields with negative polarity, the output voltage is negative. Therefore, both the amplitude and the sign of the measured electric field can be determined.

Table 22 is a comparison table that includes the most significant parameters of electric field mill front-end systems, such as the electric field range that can be measured and the sensitivity and resolution of the sensor and the power supply and power consumption, as well as the main design parameters of the field mill sensor, such as the number of vanes of the rotating electrodes, the dimensions of the sensing electrodes, the type of motor, and its rotation frequency. 

It can be observed that this work is comparable to other works found in the literature and can significantly outperform several of them in terms of power consumption, while offering the low-cost and low-area benefits of an integrated System-on-Chip. More specifically, the interface has a sensitivity of 45.75 mV/kV/m, which is combined with a measurable electric field range of ±20 kV/m. Additionally, it has a total power consumption of around 480 mW, which is significantly lower than the consumption in [4] and [26] and comparable to the consumption in [5]. In [5], lower power consumption is achieved mainly due to the low power motor used, which in this work was the main power contributor. Therefore, the use of a low-power motor or, alternatively, an intermittent style of operation of the sensor, where the motor is activated for only a small time duration each time, as suggested in [11], would lead to improved results in terms of power consumption.

## 5. Conclusions

A comparative analysis of four integrated bandpass filter implementations was conducted in XH018 process. The main goal was the development of a fully integrated field mill sensing system with high performance and an optimized silicon-to-cost ratio. Three OTA-based passive element replacement cascaded filter topologies and one implementation employing the leapfrog technique were designed, and the optimum filter performance-wise—C replacement cascaded topology—was adjusted to the specifications of the field mill analog interface application. The design of the C replacement configuration was optimized to achieve a passband of 20 Hz to 1 kHz, which remained stable at process corners, with a midband gain of about 11 dB. The final design was further improved in terms of output rms noise (~270 μV), as well as power consumption (0.38 mW) and provided a dynamic range of 47.7 dB. Post-layout simulations of the total sensor interface showed that the bandpass filter significantly diminishes the noise components caused by both the shape of the sensing electrodes and by the preamplifier. The limited dynamic range of the final filter design renders the need of an additional amplification stage. This results in an enhanced sensitivity performance of 45.75 mV/kV/m, which allows for accurate measurements even of electric fields in fair-weather conditions. The final physical design (layout) of the filter occupies an area of 130 μm × 100 μm.

The main limitation of the proposed interface is that the motor rotation should be mostly stable so that the frequency of the induced signal is within the expected range, which determines the passband specifications of the bandpass filters. The total power consumption of the sensor interface (483 mW) is low compared to other works in field mill interfacing and is almost exclusively due to the motor consumption; therefore, in applications where the field mill sensor needs to be energy autonomous, either a very low-power motor can be utilized, or a non-continuous operation of the motor should be introduced.

## Figures and Tables

**Table 1:** Field mill sensor parameters.

Parameter	Value
Number of vanes	6
Motor frequency	75 Hz
Motor type	Brushless DC
Signal frequency	450 Hz

**Table 2:** Transfer function, highpass frequency, lowpass frequency, and gain of each filter implementation.

	*Transfer Function H(s)*	$f_{c\left(\mathrm{highpass}\right)}\;$	$f_{c\left(\mathrm{lowpass}\right)}\;$	*Gain*
R simulator	$\left(\frac{1}{1+\left(\frac{RG_{m1}G_{m2}}{Cs}\right)}\right)\left(\frac{\left(G_{m3}/G_{m4}\right)}{1+\frac{Cs}{G_{m4}}}\right)\left(\frac{1}{1+\frac{Cs}{G_{m4}}}\right)$	$\frac{G_{m1}G_{m2}R}{2\pi C}$	$\frac{G_{m4}}{2\pi C}$	$\frac{G_{m3}}{G_{m4}}$
C simulator	$\left(\frac{1}{1+\left(\frac{1}{RCs}\times \frac{G_{m3}G_{m4}}{G_{m1}G_{m2}}\right)}\right)\left(\frac{\left(G_{m5}/G_{m6}\right)}{1+\frac{Cs}{G_{m6}}}\right)\left(\frac{1}{1+\frac{Cs}{G_{m6}}}\right)$	$\frac{1}{2\pi RC}\times \frac{G_{m3}G_{m4}}{G_{m1}G_{m2}}$	$\frac{G_{m6}}{2\pi C}$	$\frac{G_{m5}}{G_{m6}}$
L simulator	$\left(\frac{1}{1+\left(\frac{G_{m1}G_{m2}Rs}{C}\right)}\right)\left(\frac{\left(G_{m3}/G_{m4}\right)}{1+\frac{Cs}{G_{m4}}}\right)\left(\frac{1}{1+\frac{Cs}{G_{m4}}}\right)$	$\frac{C}{2\pi R}\times \frac{1}{G_{m1}G_{m2}}$	$\frac{G_{m4}}{2\pi C}$	$\frac{G_{m3}}{G_{m4}}$

**Table 3:** Parameter values of each filter implementation.

Cascade Topologies						Leapfrog	
Resistance Simulator		Capacitance Simulator		Inductance Simulator			
G_m2_	1.2 μA/V	G_m1_	523 μA/V	G_m2_	1.2 μA/V	G_m2_	1.2 μA/V
G_m3_	4.8 μA/V	G_m2_	1.2 μA/V	G_m3_	4.8 μA/V	G_m4_	93 nA/V
C1	11 pF	G_m3_	4.8 μA/V	C1	11 pF	G_m5_	346 nA/V
C2	12 pF	C1	1.8 pF	C2	12 pF	C1	8.3 pF
R	1 kΩ	C2	12 pF	R	1 kΩ	C2	375 pF
		R	22 kΩ				

**Table 4:** Transistor values for each OTA circuit.

OTA G_m1_	W(μm)/ L(μm)	OTA G_m2_	W(μm)/ L(μm)	OTA G_m3_	W(μm)/ L(μm)	OTA G_m4_	W(μm)/ L(μm)	OTA G_m5_	W(μm)/ L(μm)
Mn1-Mn4, Mn7	2/0.6	Mn1-Mn4	0.9/14	Mn1-Mn4	0.9/14	Mn1-Mn4	0.6/50	Mn1-	0.6/50
Mn5, Mn6	8/0.6	Mn5, Mn6	1/14	Mn5, Mn6	1/14	Mn7	0.6/50	Mn7	1.2/10
Mp2, Mp3, Mp6, Mp7	1/0.6	Mn7	1.5/14	Mn7	15/14	Mn5, Mn6	0.6/40	Mn5, Mn6	0.6/40
Mp1, Mp4, Mp5, Mp8	20/0.6	Mp1-Mp8	1/0.9	Mp1-Mp8	1/0.9	Mp2, Mp3, Mp6, Mp7	1/10	Mp2, Mp3, Mp6, Mp7	1/10
V_bias_	−1.1 V	V_bias_	−1.1 V	V_bias_	−1.1 V	Mp1, Mp4, Mp5, Mp8	1/20	Mp1, Mp4, Mp5, Mp8	1/20
						V_bias_	−1.1 V	V_bias_	−1.1 V

**Table 5:** Corner analysis of each OTA transconductance.

	OTA-Based Bandpass Filter Design G_m_ Characteristics				
	Gm Process Corners				Monte Carlo
	Corner	Slow–Slow	Typical–Typical	Fast–Fast	Mean
R simulator	G_m2_	754 nA/V	1.2 μA/V	1.8 μA/V	1.18 μA/V
	G_m3_	3.3 μA/V	4.8 μA/V	6.6 μA/V	4.77 μA/V
C simulator	G_m1_	294.7 μA/V	523 μA/V	875.1 μA/V	524.85 μA/V
	G_m2_	754 nA/V	1.2 μA/V	1.8 μA/V	1.18 μA/V
	G_m3_	3.3 μA/V	4.8 μA/V	6.6 μA/V	4.77 μA/V
L simulator	G_m2_	754 nA/V	1.2 μA/V	1.8 μA/V	1.18 μA/V
	G_m3_	3.3 μA/V	4.8 μA/V	6.6 μA/V	4.77 μA/V
Leapfrog	G_m2_	754 nA/V	1.2 μA/V	1.8 μA/V	1.18 μA/V
	G_m4_	58.9 nA/V	93.1 nA/V	140.7 nA/V	93.03 nA/V
	G_m5_	236 nA/V	346 nA/V	487.5 nA/V	345.96 nA/V

**Table 6:** Corner analysis of the power consumption.

	Power Consumption			Monte Carlo
	TM Corner	WP Corner	WS Corner	Mean
R simulator	20.1 μW	32.8 μW	11.8 μW	16.3 μW
C simulator	699.1 μW	1.25 mW	373.6 μW	705.6 μW
L simulator	20.1 μW	32.8 μW	11.9 μW	16.5 μW
Leapfrog	12.5 μW	20.5 μW	7.3 μW	9.4 μW

**Table 7:** Corner analysis of the output noise.

	Output Noise (rms)		
	TM Corner	WP Corner	WS Corner
R simulator	70.4 mV	81.3 mV	49.9 mV
C simulator	1.45 mV	1.87 mV	1.13 mV
L simulator	498.3 μV	515.4 μV	476.7 μV
Leapfrog	290 μV	384 μV	210 μV

**Table 8:** Corner analysis for the midband gain.

	Gain					
	TM Corner	WP Corner	WS Corner	@THD = −40 dB	@SNR = 0 dB	DR
R simulator	12 dB	11.3 dB	12.8 dB	V_in___peak_ = 17.2 mV	V_in___peak_ = 26.6 mV	−3.8 dB
C simulator	12.1 dB	11.4 dB	12.8 dB	V_in___peak_ = 17.5 mV	V_in___peak_ = 510 μV	30.7 dB
L simulator	12.1 dB	11.4 dB	12.9 dB	V_in___peak_ = 16.7 mV	V_in___peak_ = 176.2 μV	39.5 dB
Leapfrog	−5.93 dB	−5.89 dB	−5.96 dB	V_in___peak_ = 655.3 mV	V_in___peak_ = 850 μV	57.7 dB

**Table 9:** PVT corner analysis for the cascaded filter topologies.

	R Simulator			C Simulator			L Simulator		
Process Corner	SS	TT	FF	SS	TT	FF	SS	TT	FF
f_c(Highpass)_	8.4 Hz	20.1 Hz	44.3 Hz	20.4 Hz	20.5 Hz	21.2 Hz	8.4 Hz	20.1 Hz	44.6 Hz
f_c(Lowpass)_	5.8 kHz	10.2 kHz	17.6 kHz	5.8 kHz	10.2 kHz	17.7 kHz	5.8 kHz	10.2 kHz	17.6 kHz
Gain	12 dB	11.3 dB	12.8 dB	12.1 dB	11.4 dB	12.8 dB	12.1 dB	11.4 dB	12.9 dB
Vbias	−1.12 V	−1.1 V	−1.08 V	−1.12 V	−1.1 V	−1.08 V	−1.12 V	−1.1 V	−1.08 V
f_c(Highpass)_	14.3 Hz	20.1 Hz	27.3 Hz	24.3 Hz	20.4 Hz	17.4 Hz	14.3 Hz	20.1 Hz	27.4 Hz
f_c(Lowpass)_	8.6 kHz	10.2 kHz	12 kHz	8.6 kHz	10.2 kHz	11.9 kHz	8.6 kHz	10.2 kHz	12 kHz
Gain	12.5 dB	12 dB	11.6 dB	12.6 dB	12.1 dB	11.7 dB	12.6 dB	12.1 dB	11.7 dB
Temperature	−40 °C	0 °C	40 °C	−40 °C	0 °C	40 °C	−40 °C	0 °C	40 °C
f_c(Highpass)_	12.4 Hz	17.7 Hz	20.9 Hz	48.3 Hz	26.9 Hz	18.3 Hz	12.5 Hz	17.8 Hz	21 Hz
f_c(Lowpass)_	8 kHz	9.6 kHz	10.2 kHz	8 kHz	9.6 kHz	10.4 kHz	8 kHz	9.6 kHz	10.4 kHz
Gain	13.5 dB	12.5 dB	11.9 dB	13.5 dB	12.6 dB	12.1 dB	13.6 dB	12.6 dB	11.9 dB

**Table 10:** PVT corner analysis for the leapfrog filter topology.

	Leapfrog		
Process Corner	SS	TT	FF
f_c(Highpass)_	12.2 Hz	20.6 Hz	34 Hz
f_c(Lowpass)_	7 kHz	10.3 kHz	14.6 kHz
Gain	−5.93 dB	−5.91 dB	−5.94 dB
Vbias	−1.12 V	−1.1 V	−1.08 V
f_c(Highpass)_	18.3 Hz	20.6 Hz	22.8 Hz
f_c(Lowpass)_	9 kHz	10.2 kHz	11.4 kHz
Gain	−5.93 dB	−5.93 dB	−5.94 dB
Temperature	−40 °C	0 °C	40 °C
f_c(Highpass)_	17.8 Hz	19.8 Hz	20.9 Hz
f_c(Lowpass)_	9.3 kHz	10 kHz	10.2 kHz
Gain	−5.92 dB	−5.93 dB	−5.94 dB

**Table 11:** Bandpass filter specifications.

Filter Order	3rd
$f_{c\left(low\right)}$	20 Hz
$f_{c\left(high\right)}$	1 kHz
Gain	12 dB

**Table 12:** Narrow bandpass filter parameters.

Capacitance Simulator	
Parameter	Value
G_m1_	523 μA/V
G_m4_	93.1 nA/V
G_m5_	346 nA/V
G_m6_	46.1 μA/V
C1	450 fF
C2	10 pF
R	5 kΩ
VDD	1.8 V
VSS	−1.8 V

**Table 13:** Transistor values of each OTA design in Figure 13.

$\mathrm{OTA}\;G_{m1}$	(μm/μm)	$\mathrm{OTA}\;G_{m4}$	(μm/μm)	$\mathrm{OTA}\;G_{m5}$	(μm/μm)	$\mathrm{OTA}\;G_{m6}$	(μm/μm)
Mn1, Mn2, Mn3, Mn4,	2/0.6	Mn1, Mn2, Mn3, Mn4,	0.6/50	Mn1, Mn2, Mn3, Mn4,	0.6/50	Mn1, Mn2, Mn3, Mn4,	1/1
Mn7	2/0.6	Mn7	0.6/50	Mn7	1.2/10	Mn7	1/1
Mn5, Mn6	8/0.6	Mn5, Mn6	0.6/40	Mn5, Mn6	0.6/40	Mn5, Mn6	2/1
Mp2, Mp3, Mp6, Mp7	1/0.6	Mp2, Mp3, Mp6, Mp7	1/10	Mp2, Mp3, Mp6, Mp7	1/10	Mp2, Mp3,	6/1
						Mp6, Mp7	3/1
Mp1, Mp4, Mp5, Mp8	20/0.6	Mp1, Mp4, Mp5, Mp8	1/20	Mp1, Mp4, Mp5, Mp8	1/20	Mp1, Mp4,	24/1
						Mp5, Mp8	12/1
$V_{bias}$	−1.1 V	$V_{bias}$	−1.1 V	$V_{bias}$	−1.1 V	$V_{bias}$	−1.1 V

**Table 14:** Behavior of Gm in process corners.

Gm Process Corners				Monte Carlo
Corner	Slow–Slow	Typical–Typical	Fast–Fast	Mean
Gm1	294.7 μA/V	523 μA/V	875.1 μA/V	524.85 μA/V
Gm4	58.9 nA/V	93.1 nA/V	140.7 nA/V	93.03 nA/V
Gm5	236 nA/V	346 nA/V	487.5 nA/V	345.96 nA/V
Gm6	27.5 μA/V	46.1 μA/V	73.1 μA/V	46.07 μA/V

**Table 15:** Behavior of power consumption in corner analysis.

Power Consumption			Monte Carlo
TM Corner	WP Corner	WS Corner	Mean
280.6 μW	497.2 μW	151 μW	282.7 μW

**Table 16:** Behavior of output noise in corner analysis.

Output Noise (rms)		
TM Corner	WP Corner	WS Corner
251.3 μV	223.5 μV	268 μV

**Table 17:** Behavior of mid-band filter gain in corner analysis and dynamic range.

Gain					
TM Corner	WP Corner	WS Corner	THD = −40 dB	SNR = 0 dB	
11.3 dB	10.7 dB	11.8 dB	V_in___peak_ = 23.8 mV	V_inpeak_ = 97.9 μV	DR = 47.7 dB

**Table 18:** PVT corner analysis of low cut-off and high cut-off frequencies of the bandpass filter.

PVT Analysis				
Process Variation				
Process Corner		SS	TT	FF
f_c(Highpass)_		19.2 Hz	20.4 Hz	22.4 Hz
f_c(Lowpass)_		581.2 Hz	1 kHz	1.8 kHz
Gain		11.3 dB	10.7 dB	11.8 dB
**Voltage Variation**				
Vbias		−1.12 V	−1.1 V	−1.08 V
f_c(Highpass)_		23.2 Hz	20.4 Hz	18.2 Hz
f_c(Lowpass)_		872.4 Hz	1 kHz	1.2 kHz
Gain		11.6 dB	11.3 dB	10.9 dB
**Temperature Variation**				
Temperature	−40 °C	0 °C	27 °C	40 °C
f_c(Highpass)_	36.3 Hz	24.5 Hz	20.4 Hz	19.1 Hz
f_c(Lowpass)_	812.8 Hz	957.1 Hz	1.02 kHz	1.04 kHz
Gain	12.4 dB	11.7 dB	11.3 dB	11.1 dB

**Table 19:** Passive component characteristics of the integrated implementation.

Component	Value	Unit
R_F_	500	kΩ
Ra1	50	kΩ
Ra2	1	MΩ
Re1, Re2	2.5	MΩ
Rf (external)	2.5	MΩ
Cf (external)	10	nF

**Table 20:** Noise parameters of preamplifier op-amp.

Parameter	Value	Unit
Input voltage noise @ 450 Hz	53.65	nV/√Hz
Input current noise @ 450 Hz	2.01	fA/√Hz
Power consumption of preamplifier	404.28	μW

**Table 21:** Power consumption of the sensor interface circuits.

Parameter	Value	Unit
Power consumption of preamplifier	404.28	μW
Power consumption of filter	386.6	μW
Total power consumption	1.13	mW

**Table 22:** Table of comparison of electric field mill analog front-end systems in the literature.

Parameter	[4]	[5]	[6]	[26]	Previous Work [11]	This Work
Number of sector-shaped vanes, n	3	2	6	2 circle-shaped sensing electrodes	2	6
Sensor dimensions	11.5 cm diameter	3 cm diameter	N/A	5 cm diameter of each electrode	15.2 cm diameter	15 cm diameter
Electric field range	N/A	±150 V/m	0–80 kV/m	1.5 kV/m (high sensitivity channel) 15 kV/m (low sensitivity channel)	±20 kV/m	±20 kV/m
Sensitivity	N/A	~1 mV/V/m	48.75 mV/kV/m	N/A	45 mV/kV/m	45.75 mV/kV/m
Resolution	16 bits 2 V/m	16 bits	N/A	3 V/m (high sensitivity channel) 30 V/m (low sensitivity channel)	16 bits 0.6 V/m	16 bits 0.61 V/m
Rotation frequency, f (Hz)	33.3	60	N/A	N/A	12.75	75
Motor type	Three-phase motor Maxon EC32	N/A	Brushless DC motor	Step motor	Brushless DC motor	Brushless DC motor
Power supply	8 V motor and motor driver 5 V digital systems and sensors ±5 V analog circuitry	6 V	>4 V	N/A	3 V analog front-end supply 5 V motor supply	±1.8 V analog front-end supply 3–6 V motor supply
Power consumption	4 W	408 mW (264 mW motor consumption)	N/A	1.8 W	180.165 mW	483 mW (@3 V motor supply)

## Data Availability

Not applicable.
